# Modifying implementation strategies for integration of screening and management of mental disorders, including substance use disorders into other NCD care in Faridabad, Haryana as part of ICMR-MINDS project

**DOI:** 10.3389/fpubh.2026.1734787

**Published:** 2026-05-13

**Authors:** Yatan Pal Singh Balhara, Neha Dahiya, Tweesha Chhatbar, Parag Bhardwaj, Siddharth Sarkar, Hitesh Verma, Kuldeep Singh, Gerish Atri, Om Pal Singh Saini, Pulkit Verma, Ashoo Grover

**Affiliations:** 1 National Drug Dependence Treatment Centre (NDDTC), All India Institute of Medical Sciences (AIIMS), New Delhi, India; 2 Delivery Division, ICMR Headquarters, New Delhi, India; 3 Mental Health Services, DGHS Office, Panchkula, India; 4 Director General Health Services (DGHS), Panchkula, Haryana, India; 5 State NCD Division, NHM, Panchkula, India; 6 Data Centre, Indian Council of Medical Research Headquarters, New Delhi, India

**Keywords:** ERIC taxonomy, FRAME-IS, health systems integration, implementation strategies, low- and middle-income countries, mental and substance use disorders, non-communicable diseases, strategy adaptation

## Abstract

**Background:**

Despite the growing burden of mental disorders, including substance use disorders (MSUDs) in India, their integration within existing non-communicable disease (NCD) care remains limited. This study documents the process of modification and refinement of implementation strategies. These strategies were originally developed as part of initial models (Model M0, M1, M2, M3…). The strategies aimed to integrate MSUD screening and management within existing NCD care in the Faridabad district of Haryana, India.

**Methods:**

This was a mixed-methods implementation study. Framework for Reporting Adaptations and Modifications to Evidence-based Implementation Strategies (FRAME-IS) was used to systematically capture changes to the original set of 51 Expert Recommendations for Implementing Change (ERIC) strategies. Modifications were categorised as planned or unplanned, proactive or reactive. They were analysed based on type (substitution, addition, tailoring, or integration). Stakeholders were purposively recruited based on their role in the design, administration, or delivery of care. These included policy makers, state- and district-level health administrators, facility-level healthcare professionals, and patients/service users and caregivers. Data sources included field notes by the project field staff, digital portal, and dashboard, training reports and meetings of the project group at AIIMS, New Delhi. Additional information for the process came from the co-creation meetings. A total of 81 healthcare professionals from 16 public health facilities participated in training. Stakeholder engagement involved co-creation meetings, field-based observations, structured feedback loops, and consensus-based adaptation cycles. Qualitative data were analysed using a rapid thematic approach guided by the CFIR.

**Results:**

Nine strategies were modified substantially. These included revising professional roles, creating new clinical teams, facilitating relay of clinical data to providers, promoting network weaving, intervening with patients/consumers to enhance uptake and adherence, use mass media, conduct ongoing training, shadow other experts, and visit other sites. Workforce shortages, logistical constraints, and the absence of mass media channels were main reasons for modifications. Social incentives were introduced to enhance engagement. The refined implementation strategies were integrated into the successive models (Model M1, M2, M3…Mx). This contributed to the final implementation model development (Model Mx).

**Conclusion:**

The study highlights the importance of systematic approach and documentation for adapting implementation strategies to real-world conditions. The modified strategies intend to offer a feasible approach to integrated MSUD care in Faridabad district of Haryana. The findings indicate feasibility and system fit. Further evaluation is required to assess effectiveness and scalability.

**Clinical trial registration:**

https://ctri.nic.in/Clinicaltrials/pmaindet2.php?EncHid=MTEzMTg4&Enc=&userName=, identifier (CTRI/2024/08/072748).

## Background

Mental disorders, including substance use disorders (MSUDs), are recognised as major contributors to the global burden of disease (1). These often co-occur with other non-communicable diseases (NCDs) such as diabetes, hypertension, and cancers (2). Both these sets of disorders are influenced by similar biological, behavioural, and social factors. Depression and anxiety increase the likelihood of developing diabetes and cardiovascular disease. They also worsen outcomes once these conditions are present together. Alcohol and tobacco are known risk factors for various NCDs. Long-term NCDs also contribute to ongoing stress, physical limitations, and financial strain. These can increase the risk of MSUDs (3). MSUDs further worsen NCD outcomes through continued exposure to shared risk factors (4). Also, they reduce treatment adherence and self-care.

The dual burden of NCDs and MSUDs presents challenges to the public health system in India (5, 6). Currently, services for MSUDs and other NCDs are delivered through parallel vertical programmes with limited integration. These programmes include the National Mental Health Programme (NMHP) and National Programme for Prevention and Control of NCDs (NP-NCD) (7, 8). This leads to missed opportunities for early identification and comprehensive and continued care (9).

The need and rationale to integrate MSUD care within the existing NCD care framework in Faridabad district of Haryana state has been highlighted (9). However, the complexities involved, and presence of systemic, organisational, and implementation-level challenges limit such attempts. The Implementation Research Study on Integration of Screening and Management of Mental and Substance Use Disorders with Other Non-Communicable Diseases (ICMR-MINDS) project was conceptualised in response to this critical public health need. It aims to develop and evaluate a feasible, scalable, and sustainable model for integrating MSUD care within existing NCD services in the public health system in India (10). The study has been identified as a National Health Research Priority by the Indian Council of Medical Research (ICMR) (11).

The formative phase included desk reviews, SWOT analysis, care pathway mapping, and stakeholder interviews (IDIs and FGDs). The objective was to evaluate system readiness and identify both facilitators and barriers to integration. Implementation science-based approach including the Consolidated Framework for Implementation Research (CFIR), Implementation Mapping (IMap), Intervention Mapping (IM), Implementation Research Logic Model (IRLM), RE-AIM framework was used to execute the proposed integration of MSUD care into the existing NCD care in the Faridabad district of Haryana state in India (12). Model M0 was the initial implementation model developed to integrate screening and management of MSUD into existing NCD care in the public health system of Faridabad district of Haryana. It defined clear roles, actions, and workflows across the health facilities. It also linked each strategy to specific change objectives and determinants. It included 51 implementation strategies, five interventions (innovations), implementation materials (practical tools and protocols), and indicators to assess fidelity, feasibility, and acceptability. As a pre-implementation blueprint, Model M0 translated theory and field evidence into an operational plan for pilot testing and future scale-up in public health settings in Faridabad district of Haryana. This phase has been completed.

Phase II of the project involved Model Optimisation. The initial model (M0) was piloted in a single block. It was refined iteratively leading to subsequent interim models M1, M2, M3…through real-world implementation. This process would eventually lead to the development of the optimised model (Model Mx).

This article documents the process and outcomes of modifications made to the implementation strategies that were created for this project. This article attempts to provide a replicable blueprint for conducting and documenting implementation strategy modifications in similar integration efforts. This is done with an intent to enhance the transparency, reproducibility, and contextual relevance of implementation science in low- and middle-income countries (LMIC) in Southeast Asia and other regions. A dearth of similar efforts in the literature makes such an attempt imperative.

## Methods

### Aim

The aim of this article was to document the process and outcomes of the modifications to the implementation strategies that were developed as part of implementation Model M0 (and subsequent interim models M1, M2, M3…) for integration of screening and management of MSUD care into other NCD care in the Faridabad District of Haryana state. This will contribute to creation of implementation strategies for the Model Mx.

### Setting and study context

The study was conducted in the Faridabad district in the Indian state of Haryana. The district has a population of more than 2,400,000. The district has more than 116 public health facilities across different levels, including Ayushman Arogya Mandirs- Sub Centres (AAM-SC), Ayushman Arogya Mandirs- Primary Health Centres (AAM-PHC), Community Health Centres (CHC), Sub-Divisional (District) Hospital, and the District Hospital. The Faridabad district was selected in consultation with the state health authorities. The district’s proximity to the host institution of the project team was an important consideration. This was critical given the need for extensive field activities. Faridabad includes both rural and urban populations. It was identified as one of the weaker-performing districts in Haryana on several indicators in prior health systems research (13). The district scored 0.49 out of 1 on the Health System Performance Index (HSPI). It had one of the lowest densities of core public health workers in the state. Gaps were observed in the readiness of public health facilities, including inadequate availability of essential equipment, medicines, supplies, and functional infrastructure. The health information system was limited by poor data quality, completeness, and usability. Additionally, service utilisation was low. This offered an opportunity to test the model in a challenging implementation environment. Demonstration of success in such a setting was expected to support wider adoption across the state.

The Model M0 was rolled out in the Tigaon block. The Tigaon block of Faridabad district was purposively selected. The block includes a full set of public health facilities including one CHC, multiple AAM-PHC and AAM-SC. This allowed implementation across all levels of health facilities within a single administrative unit. The Tigaon block had typical district characteristics. These included variable staffing patterns, high outpatient volumes, and infrastructural constraints. These features made Tigaon an appropriate setting for identifying feasibility challenges and facilitators. This made the adaptations likely to be relevant and transferable to other blocks within the district.

**Figure 1:**
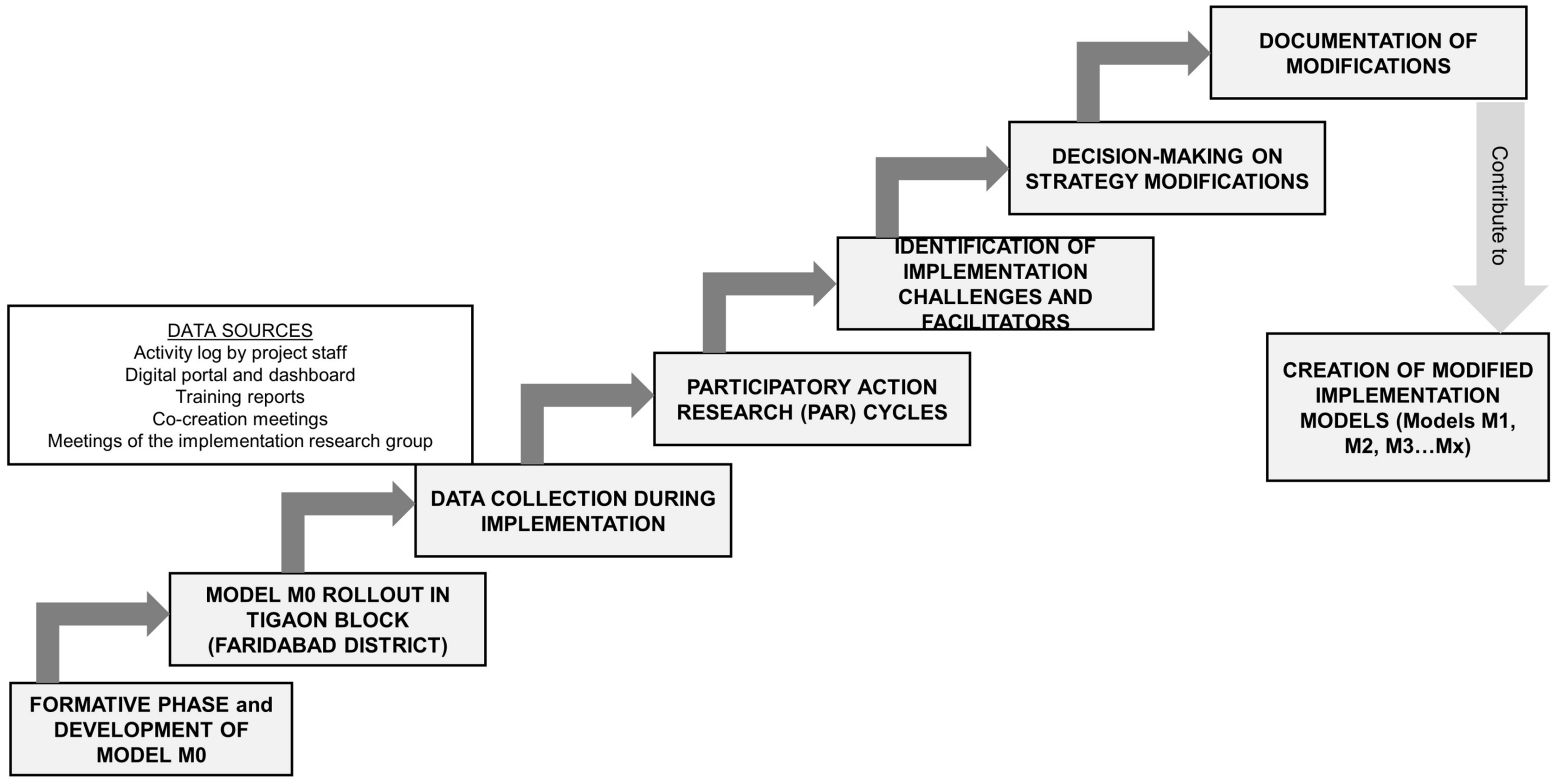
Steps of the process followed for the study.Steps of the process followed for the study.

The process followed for the study is summarised in Figure 1 and described below.

### Data collection, management and analysis

#### Data collection

**Figure 2:**
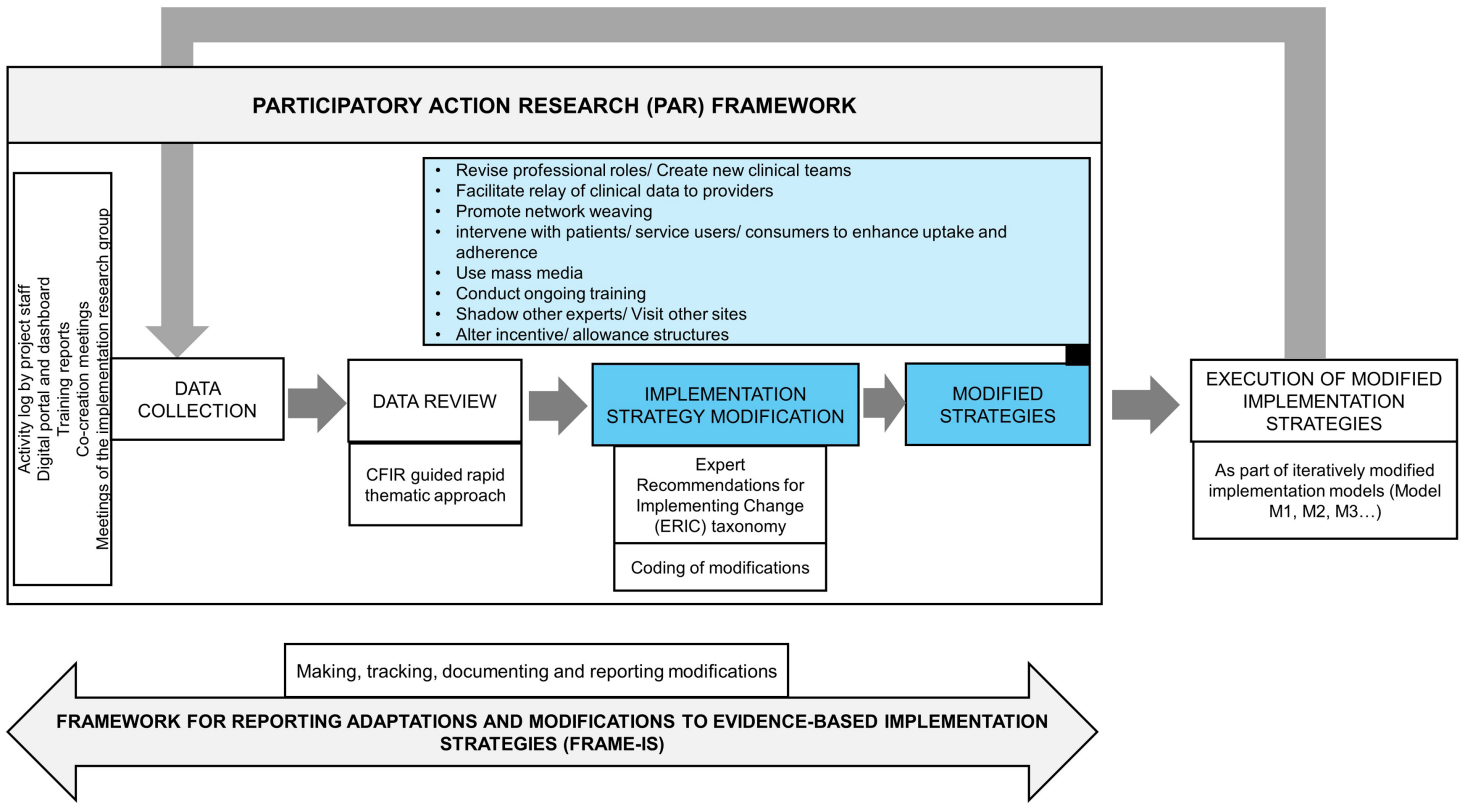
Frameworks used and the steps involved in modification of the strategies during the refinement of Model M0 and subsequent interim models M1, M2, M3.

The data sources used included the field notes by the project field staff, digital portal and dashboard (based on ICMR-MINDS CDSS inputs), training reports and meetings of the project group at AIIMS, New Delhi. In addition, the co-creation meetings conducted with the various stakeholders offered valuable information for the process. These meetings also offered opportunities to share the modifications with the stakeholders and get their concurrence (Figure 2).

##### Activity log by the project staff

Data collection Google Sheets were created for the project field staff to keep a log of the activities carried out during the implementation of Model M0 (and subsequent iterations). Field staff were affiliated with the ICMR-MINDS Haryana site and had backgrounds in psychology, social work and medical science. Their role was to support facility visits, observe routine service delivery, assist with training and co-creation activities, document implementation challenges and facilitators, and communicate operational issues to district authorities and the central project team. Field staff did not provide clinical care. The field staff received structured training on the purpose, format, and use of the activity logs. Training covered definitions of log fields, examples of common implementation issues, and guidance on documenting observations, stakeholder feedback, actions taken, and unresolved concerns. Staff were instructed to complete entries on the same day as field visits and to record information descriptively rather than interpretively. Senior members of the project team reviewed logs at regular intervals to ensure completeness and consistency. They also provided corrective feedback when needed.

Each project staff member maintained a daily log of their activities. They recorded the health facility visited, the Healthcare Professionals (HCPs) visited, the cadre of HCP, doubts/concerns raised by the HCP; solutions offered; suggestions made by the HCPs; other issues that were discussed; specific activities that were carried out; issues that remained unaddressed/unresolved; possible solutions for unresolved issues; other suggestions; and the date for which next visit was planned. This helped with consistent and standardised data collection, monitoring and troubleshooting, enhanced accountability and reflection, informed decision-making, collaboration and learning, and improved planning. A total of 381 field visit notes were made. The activity logs were used solely to document implementation processes. Access to the logs was restricted to authorised members of the research team through password-protected accounts.

##### Digital portal and dashboard

The information from the activity logs was supplemented with the data from the digital portal and dashboard that was created as part of the project. The dashboard offered real-time data on the adoption of the interventions by the HCPs. Information was available on the number of cases screened, assessed, treated and referred by each HCP at each of the health facilities. In addition, the cumulative numbers of the patients that were screened positive and diagnosed for different MSUD was also available. However, identifiable details of any of the patients were not accessible to ensure privacy.

##### Training reports

Feedback was collected from the participants after the completion of the trainings that were conducted as part of the project. These included training of Medical Officers (MOs), Community Health Officers (CHOs), Auxillary Nurse Midwife (ANM), nursing staff and Accredited Social Health Activist (ASHA). A total of 21 trainings were conducted in which 81 HCPs were trained. The feedback from the participants was also another source of data. This supplemented the data from the daily log maintained by the project staff for the field visits that have been described above.

##### Co-creation meetings

Mendelow’s Matrix (Stakeholder Mapping Matrix) was used to analyse and categorise actors (stakeholders) based on their level of power and interest in the health system (14). Mendelow’s Matrix categorises the stakeholders into four groups including key players (stakeholders with high power, high interest), keep managed (stakeholders with high power, low interest), keep informed (stakeholders with low power, high interest), and monitor (stakeholders with low power, low interest). High-power, high-interest stakeholders included senior state- and district-level decision-makers such as the Chief Secretary and Additional Chief Secretary (Health), Director National Health Mission (NHM), Director General of Health Services (DGHS), state and district nodal officers for NCD and mental health, WHO NCD consultant, Civil Surgeon, district programme officers, and representatives from ICMR headquarters. Stakeholders with high power but relatively lower interest included health facility administrators and monitoring and evaluation officers. Frontline implementers (MOs, CHOs, Officers, ANMs nursing staff, ASHA), and patients/service users and caregivers were classified as low-power but high-interest stakeholders. Community members, non-governmental organisations, and advocacy groups were categorised as low-power, low-interest stakeholders.

The recommendations of the International Association for Public Participation (IAP2) were used to enhance stakeholder engagement (15). IAP2 provides a set of core recommendations for effective stakeholder engagement through its Public Participation Spectrum. It outlines five levels of engagement (inform, consult, involve, collaborate, and empower). This helps ensure that participation is aligned with the goals of both decision-makers and the affected communities.

A total of 14 co-creation meetings were conducted with various stakeholders over a period of 5 months. These meetings were organised at the level of state, district, and various health facilities. Participants for co-creation meetings were selected using purposive criteria based on their direct role in design, administration, or delivery of health services in Faridabad district. Selection focused on individuals with decision-making authority at the state and district levels and those responsible for routine implementation at health facilities. Participants were invited through official channels to ensure representation across system levels and professional roles. Group dynamics and role hierarchies were considered during meeting planning and facilitation. Meetings were structured to separate system-level decision-making from operational problem-solving when needed. This approach supported participation and practical decision-making.

The participants in these meetings included Chief Secretary of Haryana, Additional Chief Secretary Health Haryana; State Programme Officers (NCD, MH); Civil Surgeon (Chief Medical Officer), Faridabad District; Deputy District Civil Surgeon cum Nodal Officer for National Mental Health Programme, Faridabad District; DGHS Haryana; Director NHM, Haryana; Senior Medical Officer, CHC Tigaon, Medical Officers, CHOs, ANMs, Nursing staff, ASHAs from Tigaon block; psychiatrists working at the Comprehensive Rural Health Services Project (CRHSP), Ballabgarh; patients/service users and caregivers; DG ICMR; Head, Delivery Division, ICMR Hqrs; Programme officer (Mental Health), ICMR; ICMR techincal team, AIIMS, New Delhi project team.

##### Meetings of the project group

Since the beginning of the project, members of the project team, including the project principal investigator and project staff (Project Research Scientist Medical (one in number), Project Research Scientist Non-Medical (one in number), Project Technical Support (four- six in number)) met weekly to discuss implementation. Topics covered included reviewing digital portal and dashboard data, training of the HCPs, data on screening, diagnosis, and management of MSUDs, uptake of interventions at different health facilities and by different HCPs; discussing barriers and facilitators to implementation; and identifying changes that could be made to support implementation.

#### Participatory action research approach

A participatory action research (PAR) approach was used to continuously monitor, refine and modify strategies based on real-time field insights. This approach has been widely used in research aimed at strengthening health systems and services (12, 16, 17).

PAR was operationalised through iterative cycles The PAR cycle included implementing Model M0 (and subsequent iterations M1, M2, M3…) in routine services (action); watching how it worked and noting problems (observation); discussing findings in team and stakeholder meetings (reflection); making practical changes to strategies (adaptation).

The data sources used included the activity logs by the project field staff, digital portal, and dashboard, training reports and meetings of the project group at AIIMS, New Delhi. In addition, the co-creation meetings with the various stakeholders offered valuable information for the process.

Project field staff maintained structured daily logs using a Google Sheet-based tool. These logs recorded information as described above. This documentation helped create a dynamic feedback loop between implementation and learning. These records provided understanding of the patterns in concerns raised by HCPs. These also helped with identification of common implementation barriers and facilitators. This information was supplemented by the data from the digital portal and dashboard, training reports, co-creation meetings and meetings of the project group at AIIMS, New Delhi. This participatory process ensured that implementation was responsive to the real world context. The strategies were modified iteratively accordingly.

CFIR guided rapid thematic approach was used to analyse the qualitative data from meeting notes, field logs, and interview summaries. Findings were reviewed by the project team periodically. These were discussed with district and state stakeholders to reach practical consensus on adaptations. Decision-making followed an iterative consensus-building process. This involved the health facility-level implementers, district and state health authorities and AIIMS New Delhi project team. The proposed modifications were evaluated against three guiding questions. These included the following: Does the adaptation improve feasibility or acceptability without compromising core functions?; Does it strengthen alignment with health system capacity and workflows?; Will it support scalability and sustainability at district level? Modifications meeting these criteria were included. The decisions about adaptation were documented using FRAME-IS and validated through consensus with district and state stakeholders. This was done to avoid potential bias.

#### Tracking, documenting and reporting modifications

The Framework for Reporting Adaptations and Modifications to Evidence-based Implementation Strategies (FRAME-IS) was used (18). This ensured systematic tracking and transparency of documentation and reporting these modifications. FRAME-IS is specifically designed to guide the documentation of changes made to implementation strategies. It is a modular approach consisting of four core and three optional components. The information is recorded using these. This includes what was modified, the nature and rationale of the modification, when and how it occurred, and who was involved in the decision-making process (19). This framework has been adopted across diverse implementation contexts to track modifications systematically (20–22). It has been found useful in public health research. It has been used to describe adaptations in outreach and intervention strategies designed for population-level impact (23).

**Figure 3:**
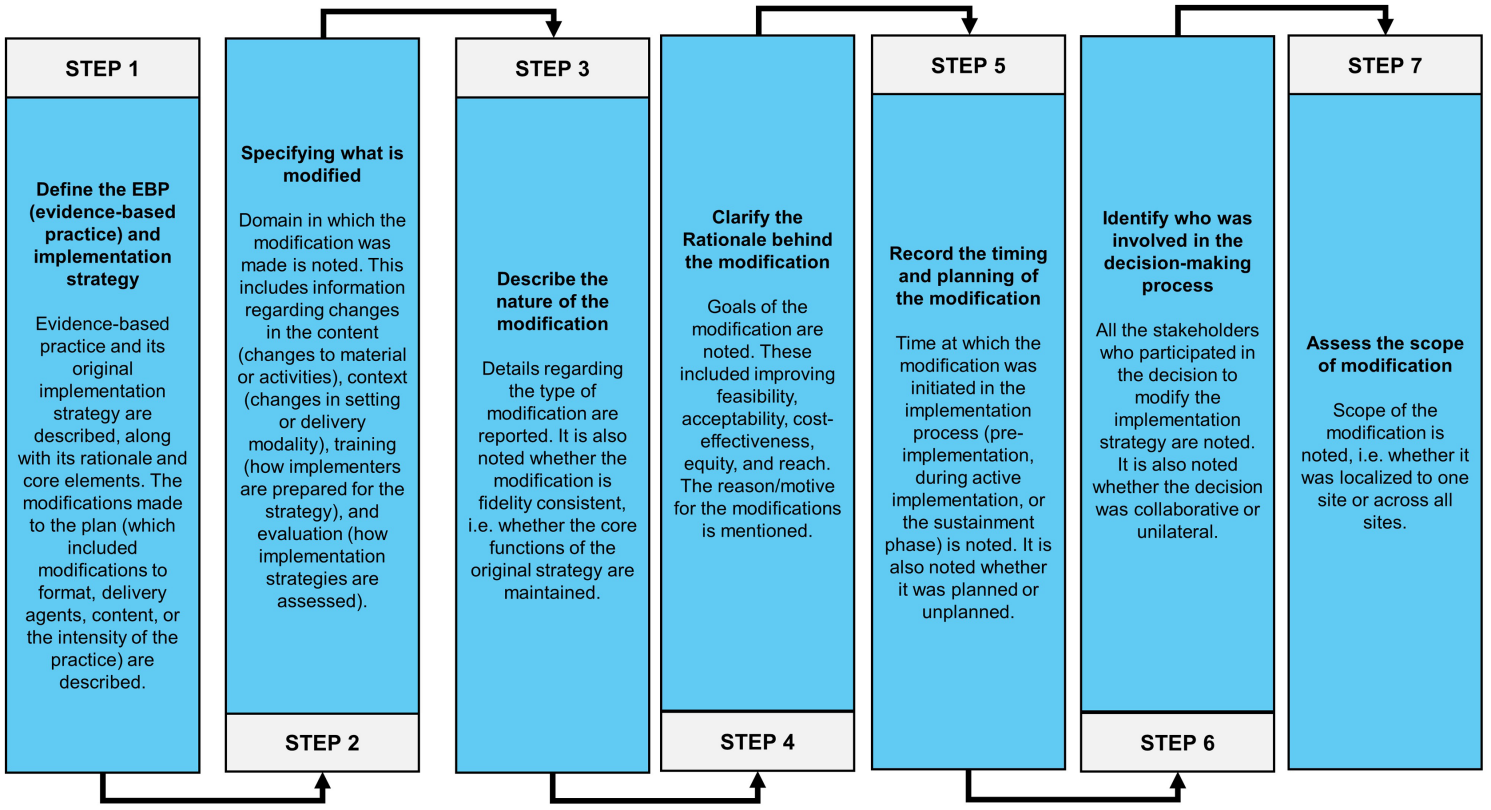
Steps of the Framework for Reporting Adaptations and Modifications to Evidence-based Implementation Strategies (FRAME-IS).

FRAME-IS recommends a series of seven steps (Step 1 – Step 7) to document the modifications. These steps are summarised in Figure 3. These steps were followed to document each modification that was made to the implementation strategies.

#### Choice of taxonomy

Expert Recommendations for Implementing Change (ERIC) taxonomy was used for modified implementation strategies (24). This choice was guided by the fact that earlier ERIC was used to develop the initial implementation strategies (34). It also facilitated the capture of different implementation strategies for integration of screening and management of MSUD into other NCD care in the Faridabad District of the Haryana state. In addition, it also helped to identify and document any deviations from the core elements.

#### Categorising modifications

Two categories were used for coding modifications. These included proactive modifications (changes in response to anticipated barriers) and reactive modifications (changes due to unanticipated barriers during the implementation process). In addition, the modifications were categorised as planned modifications (modifications that are decided using a systematic process) and unplanned modifications (modifications without a systematic process) as used by Schoenthaler et al. (19).

Due to space constraints, we have reported the substantive (core-function) modifications to the implementation strategies in this article. The rest are categorised as refinement (form-preserving) modifications and have not been reported, although a similar process was followed for these as well. We followed the Standards for Quality Improvement Reporting Excellence (SQUIRE) 2.0 guidelines for reporting new knowledge about how to improve healthcare (attached as a Supplementary file) (25).

## Results

### Modifications to the implementation strategies

A total of 51 ERIC strategies were identified to be part of Model M0. Modifications were made across nine implementation strategies as part of the refinement of the Model M0 (and other subsequent interim models M1, M2, M3…) to reach Model Mx. Tables 1-7 summarise the modifications that were made to the implementation strategies based on FRAME-IS for the integration of screening and management of MSUD care into the existing care for other NCDs in the Faridabad District of the Haryana state. Substitution of elements, addition of elements, tailoring of strategy, and integration were used for making modifications to the strategies.

**Table 1:** Modifications to implementation strategy 1 and 2 for integration of screening and management of mental disorders, including substance use disorders (MSUD) care within the existing other non-communicable diseases (NCD) care in Faridabad district of Haryana state based on FRAME-IS.

Step 1: Strategy	Strategies: Revise professional roles and Create new clinical teams Specific action(s): Develop a proposal for new staffing arrangements (number and roles)
Step 2: What is modified?	Modification was made to the content and context (population) of the strategy. The content and context (who does the work and who provides specialist care) were changed. At health facilities where Community Health Officers (CHO) were not available, ANMs and nursing staff were trained to do screening, basic management, and referrals for MSUD using the ICMR-MINDS CDSS. Also, instead of waiting for new psychiatrists to be appointed, psychiatrists from CRHSP Ballabgarh were identified to provide specialist care for referred patients.
Step 3: Nature of modification	Substitution of elements – ANMs and nursing staff at facilities with no CHOs were trained on the screening, management and referral workflow. Specific action(s) – At the health care facilities where there were no CHOs appointed, ANMs and nursing staff were trained on the screening, management and referral workflow. In addition, they were trained for screening and intervention for MSUD.
	Addition of elements – identifying mental health professionals to offer specialised services for MSUD to patients who were referred by CHOs/MOs following screening and assessment. Specific action(s) – Psychiatrists working at the Comprehensive Rural Health Services Project (CRHSP), Ballabgarh, were identified to offer specialised services for those patients who were referred by the CHOs/MOs following screening/assessment.
Step 4: Goal and rationale	Goal: To make available screening, intervention and referral services at those health facilities where CHOs were not appointed. To make specialised services for MSUD available within the district. Rationale: A complete revision of the professional roles was delayed. It was not possible to create a new staffing arrangement as the change in staffing arrangement in the health system required approval at the highest level of bureaucracy. It was a time-intensive process and would have required major changes. In addition, the creation of new clinical teams was also not feasible since the appointment against the vacant posts of CHO was delayed due to administrative reasons. Appointment of a psychiatrist at the district hospital was delayed due to administrative reasons. Level: organisational level (available staffing and resources)
Step 5: When was the modification initiated?	The modification was initiated during the implementation phase, after CHO and MO training across the Tigaon block was completed, and they had started offering the project interventions at their respective facilities.
Step 6: Who participated in the decision?	Chief Medical Officer, Faridabad District; Deputy District Civil Surgeon cum Nodal Officer for National Mental Health Programme, Faridabad District; Roster in-charge of ANMs and nursing officers, Faridabad district; Senior Medical Officer, Tigaon Block, ANMs/nursing officers, Psychiatrists working at the Comprehensive Rural Health Services Project (CRHSP), Ballabgarh. The decision was taken collaboratively.
Step 7: How widespread is the modification?	In the context of CHOs, the modification has been implemented for specific centres where CHOs have not been appointed. In the context of psychiatrists, the modification has been implemented at the second referral level. It would impact forward referrals at all facilities. The modification will remain in place until the appointment of the CHOs and psychiatrist at the vacant posts in the district.

The AIIMS, New Delhi Project team was also part of the discussions around the modifications for the strategies. AIIMS, All India Institute of Medical Sciences; HCP, Health care professionals; ICMR, Indian Council of Medical Research.

The first and second modified strategies were revising professional roles and creating new clinical teams. The content and context (population) of the strategy were modified. The ANMs and nursing staff at healthcare facilities where CHO have not been appointed were trained to screen, manage and refer patients with MSUDs. The goal of this adaptation was to make screening, management and referral services available at facilities where CHOs were not appointed. The psychiatrists working at the CRHSP (Comprehensive Rural Health Services Project) Ballabgarh were identifed to attend to the referrals made to the psychiatrists. The goal of this adaptation was to make specialised services for MSUD available and accessible within the public health system in the Faridabad district. The rationale for these two modifications was that a complete revision of professional roles was not feasible due to the process being time-intensive and requiring approval at the highest level of bureaucracy. Moreover, the appointments for CHOs and the psychiatrist post vacancies were delayed (Table 1).

The third modified strategy was to facilitate relay of clinical data to providers. The context (population) of this strategy was modified, whereby the dashboard of the platform (viewer-only rights) developed as part of the project was made accessible to state-level health system leaders from Haryana. This allowed them access to real-time updates on the adoption of interventions (ICMR-MINDS CDSS) by health care professionals. It also provided them with opportunities to take necessary steps to mitigate the slow rate of adoption of interventions by HCPs. The change was initiated after the request was received from the health system leaders during a project progress update briefing, post 8 weeks of implementation. The decision was made after approval from the ICMR technical team that was responsible for authorising access to the dashboard (Table 2).

**Table 2:** Modifications to implementation strategy 3 for integration screening and management of mental disorders, including substance use disorders (MSUD) care within the existing other non-communicable diseases (NCD) care in Faridabad district of Haryana state based on FRAME-IS.

Step 1: Strategy	Strategy: Facilitate relay of clinical data to providers Specific action(s): Assess existing/non-existing Health Information Technology (HIT) systems
Step 2: What is modified?	The context (population- who can see the information) of the strategy was modified. Earlier, only the project team lead could see the digital dashboard. Now, state-level health system leaders were also given viewer-only access to see summary data (without patient names).
Step 3: Nature of modification	Addition of elements: providing viewer rights to deidentified and aggregated data on the digital dashboard to state health system leaders Access to the dashboard of the digital platform developed as a project intervention was also provided to the health system leaders at the state level in Haryana. They were provided viewer-only rights.
Step 4: Goal and rationale	Goal: To offer real-time updates on the use of ICMR-MINDS CDSS by the health care professionals to health system leaders. Rationale: By accessing the dashboard of the project digital platform, the state health system leaders can monitor uptake of the interventions (ICMR-MINDS CDSS) by HCPs in the Faridabad district. This would provide them with a real-time status update, as well as opportunities to take necessary remedial actions in case of a delay in the adoption of interventions by HCPs. Level: Health system level (health system leaders)
Step 5: When was the modification initiated?	The modification was initiated in the implementation phase. It was done after receiving the request from the health system leaders during an interaction as part of a project progress update briefing. This occurred approximately 8 weeks after the implementation of in the Tigaon block.
Step 6: Who participated in the decision?	ICMR Technical Team. The decision was taken unilaterally.
Step 7: How widespread is the modification?	The modification will be implemented for the remainder of the project.

The AIIMS, New Delhi Project team was also part of the discussions around the modifications for the strategy. AIIMS, All India Institute of Medical Sciences; HCP, Health care professionals; ICMR, Indian Council of Medical Research.

The fourth modified strategy was promoting network weaving through meetings, collaborative learning sessions, sharing experiences, problem-solving and peer support. The context (format & setting) of network-weaving was amended from formal to informal interaction sessions. It was aimed at allowing the adoption of the intervention by HCPs and facilitating support during the process of adopting the intervention. The reasoning for this change was that planning dedicated meetings for this purpose was not possible due to the daily schedule of HCPs. Additionally, there was limited scope to expand the agenda of the pre-planned meetings due to time constraints. Brief informal interactions amongst HCPs provided them with a space and opportunity to discuss issues related to adoption of the interventions (Table 3).

**Table 3:** Modifications to implementation strategy 4 for integration of screening and management of mental disorders, including substance use disorders (MSUD) care within the existing other non-communicable diseases (NCD) care in Faridabad district of Haryana state based on FRAME-IS.

Step 1: Strategy	Strategy: Promote network weaving Specific action(s): Organise meetings, collaborative learning sessions to discuss issues and updates, share experiences, problem-solving, and support one another
Step 2: What is modified?	Modification was made to the context (format and setting- how and where meetings happened) of the strategy. Instead of holding formal, planned meetings, the team used informal discussions that took place during routine work or existing meetings at health facilities.
Step 3: Nature of modification	Tailoring of strategy: organising informal interaction sessions The modality to implement network weaving was amended from formal interaction sessions to informal interactions.
Step 4: Goal and rationale	Goal – to allow the HCPs to support the adoption of the interventions by their colleagues, as well as receive support from them during the process of adoption of these interventions. Rationale – Given the daily schedule of the HCPs, it was not possible to plan dedicated meetings for them for this purpose. The busy agenda of the pre-existing monthly meetings offered limited opportunities to introduce additional items. Informal interactions amongst the HCPs offered a practical solution. While such interactions were brief and limited, they offered some opportunities for HCPs to interact on themes related to the project. Level: Practitioner (HCP) level
Step 5: When was the modification initiated?	The modification was initiated in the implementation phase. The modification was rolled out after the training of the HCPs. When attempts were made to organise the interaction sessions, there were no opportunities to schedule them.
Step 6: Who participated in the decision?	HCPs. The decision was taken collaboratively.
Step 7: How widespread is the modification?	The modification has been implemented for the entire network system (Faridabad district) and will be rolled out for the remainder of the project.

The AIIMS, New Delhi Project team was also part of the discussions around the modifications for the strategy. AIIMS, All India Institute of Medical Sciences; HCP, Health care professionals; ICMR, Indian Council of Medical Research.

The fifth adaptation was about involvement of patients/service users/caregivers. Initially, the relevant stakeholders were mapped out using Mendelow’s matrix based on influence, power, authority and importance to initiate stakeholder groups. Patients/service users and caregivers were initially proposed to be included in these workgroups. The context (format) of the strategy was modified. The goal of this modification was to ensure that patients/service users/caregivers’ perspectives and opinions were incorporated in the implementation process. The adaptation was made since patients/service users and care givers found the travel to the workgroup meeting location and the extended waiting periods between meetings to be inconvenient (Table 4).

**Table 4:** Modifications to implementation strategy 5 for integration of screening and management of mental disorders, including substance use disorders (MSUD) care within the existing other non-communicable diseases (NCD) care in Faridabad district of Haryana state based on FRAME-IS.

Step 1: Strategy	Strategy: Intervene with patients/ service users/consumers to enhance uptake and adherence Specific action(s): Identify relevant stakeholders; Map stakeholders by their influence, power, authority, and importance in relation to implementation using Mendelow’s matrix; Activate stakeholder groups
Step 2: What is modified?	Modification was made to the context (format- how patients/ service users and caregivers were involved) of the strategy. Instead of asking patients and caregivers to attend group meetings and advisory boards, their views were collected through one-to-one interviews at times and places convenient for them.
Step 3: Nature of modification	Substitution: patients/service users/caregivers will be consulted for perspectives and suggestions through in-depth interviews. Inclusion of the patients/service users and caregivers in the advisory board and workgroups was planned in the beginning. After modification, patients/service users and caregivers’ perspectives and suggestions were planned to be captured using IDIs.
Step 4: Goal and rationale	Goal – to ensure that the perspectives and opinions of the patients/service users and caregivers are incorporated in the implementation process. Rationale – the patients/service users and caregivers did not find it convenient to attend the workgroup meetings as they had to wait for extended time periods and were also expected to travel to another location that was different from the health facility where they were receiving services. Level: patient/service user and care givers level
Step 5: When was the modification initiated?	The modification was initiated during the initial weeks of the implementation of the model. after getting aware of the functioning of the health facilities and the timing and venue of the workgroup meetings.
Step 6: Who participated in the decision?	Patients/service users and caregivers. The decision was taken collaboratively.
Step 7: How widespread is the modification?	The modification has been made for all patients/caregivers/service users and will be implemented for the remainder of the project.

The AIIMS, New Delhi Project team was also part of the discussions around the modifications for the strategy. AIIMS, All India Institute of Medical Sciences; HCP, Health care professionals; ICMR, Indian Council of Medical Research.

The sixth modification use of mass media to disseminate information about the implementation. The context (format, setting, and personnel) of the strategy was modified, whereby the information on the availability of MSUD services was relayed through awareness sessions and interactions with patients/service users and caregivers. The ASHAs were also encouraged to disseminate information regarding MSUD services during population-based screening for NCDs and home visits to deliver other health services. Information, Education and communication (IEC) material was also displayed at the health facilities, where it was easily visible to the visiting patients/service users and caregivers. The revision ensured that the information about the availability of MSUD services reaches potential beneficiaries. This strategy enabled the timely dissemination of information despite the lack of available mass media channels, the anticipated delays in creating a new one, and the unavailability of necessary resources to do so (Table 5).

**Table 5:** Modifications to implementation strategy 6 for integration of screening and management of mental disorders, including substance use disorders (MSUD) care within the existing other non-communicable diseases (NCD) care in Faridabad district of Haryana state based on FRAME-IS.

Step 1: Strategy	Strategy: Use mass media Specific action(s): Spread information about the implementation using IEC material
Step 2: What is modified?	The context (format, setting, and personnel- how, where, and by whom information was shared) of the strategy was modified. Instead of using mass media information about MSUD services was shared through awareness sessions at health facilities, direct talks with patients and caregivers during consultations, ASHAs during home visits and NCD screening, and posters and IEC materials displayed at health facilities.
Step 3: Nature of modification	Substitution: use of awareness sessions and IEC material to disseminate information about MSUD services being provided at health facilities instead of the use of mass media. Instead of using the mass media, the information on the availability of MSUD services at the health facilities was disseminated through the awareness sessions and by interaction with the patients/service users and caregivers during the consultations for NCD care. Additionally, the ASHAs were encouraged to spread the information during the population-based screening for NCDs and home visits they made to deliver other health services in the community. IEC material was also posted at the health facilities in locations where it was easily visible to the visiting patients/service users and caregivers, as planned initially.
Step 4: Goal and rationale	Goal – to ensure that the information about the availability of the MSUD services in the Tigaon block reaches the potential beneficiaries. Rationale – there were no existing mass media communication channels that could be utilised for this purpose. Creation of such channels was anticipated to be time-intensive and would have delayed the dissemination of the information. Additionally, it would have required additional resources, which were not available. Level: organisational level (lack of existing mass media channels)
Step 5: When was the modification initiated?	The modification was initiated during the initial weeks of the implementation of the model.
Step 6: Who participated in the decision?	HCPs. The decision was taken collaboratively.
Step 7: How widespread is the modification?	The modification has been made for the entire public health system (Faridabad district), and will be implemented for the remainder of the project.

The AIIMS, New Delhi Project team was also part of the discussions around the modifications for the strategy. AIIMS, All India Institute of Medical Sciences; HCP, Health care professionals; IEC, Information, Education and Communication; ICMR, Indian Council of Medical Research.

The seventh modification was regarding the strategy of conducting ongoing training, which involves conducting induction, follow-up, and refresher training on use of interventions. The initial plan was to conduct the training on screening, assessment and management of MSUD, and the training on the use of ICMR-MINDS CDSS separately. The content of the strategy was modified, whereby the training was modified to integrate these two into one module. The context (setting) of the strategy was also modified, i.e., the venue for training healthcare professionals was shifted from AIIMS New Delhi to health facilities. These changes ensured that all HCPs could participate in the training, It also synchronised the learning of the two modules, and reduced the duration for which HCPs were required to be away from their routine work to minimise disruption of routine services at health facilities. The rationale for the changes was the logistical constraints related to the participants’ travelling to AIIMS New Delhi, which is at a distance of around *29* km from the Faridabad district hospital. The distance is even greater for some health facilities (more than 60 km) situated in the Faridabad district. The long commute and traffic congestion led to some of the HCPs arriving late and caused considerable inconvenience to the HCPs. In addition, coming to AIIMS, New Delhi, for training meant staying away from the health facility. Due to this, the HCPs faced additional challenges in attending training due to competing responsibilities at the workplace (Table 6).

**Table 6:** Modifications to implementation strategy 7 for integration of screening and management of mental disorders, including substance use disorders (MSUD) care within the existing other non-communicable diseases (NCD) care in Faridabad District of Haryana state based on FRAME-IS.

Step 1: Strategy	Strategy: Conduct ongoing training Specific action(s): Conduct induction, follow up and refresher trainings on use of interventions
Step 2: What is modified?	The content and context (setting- what was taught and where training happened) of the strategy were modified. Two separate trainings (one on MSUD care and one on ICMR-MINDS CDSS use) were combined into one training, and the training location was shifted from AIIMS New Delhi to local health facilities in Faridabad.
Step 3: Nature of modification	Integration: combining MSUD training and ICMR-MINDS CDSS training into one. Substitution: shifting training venue from AIIMS New Delhi to health facilities in Faridabad. Initially, it was planned to conduct training on screening, assessment and management of MSUD and training on the use of ICMR-MINDS CDSS separately. The training agenda was modified to integrate the two.
Step 4: Goal and rationale	Goal: To ensure that all HCPs were able to participate in the training workshop with the least disruption of their other activities. To synchronise the learning on screening, assessment and management of MSUD with the use of respective sections of the ICMR-MINDS CDSS. To reduce the number of days for which the HCPs were required to be away from their routine work to minimise the disruption of routine service at the health facilities. Rationale: To begin with, the training workshops were conducted at AIIMS, New Delhi. The venue was located at a distance of around 29 kms from the Faridabad district hospital (some facilities situated more than 60 kms away). Some of the participants would get late reaching the training venue. Others reported an inability to attend due to competing responsibilities at the workplace. The ICMR-MINDS CDSS incorporated workflows that were included in the training on the screening, assessment, intervention and referral for MSUD. The integration of the two trainings avoided duplication of efforts. Level: implementer level (Health care professionals)
Step 5: When was the modification initiated?	The modification was made during the implementation phase. The modification was made after three training workshops (2 for MOs and 1 for CHOs) were completed. The modification was done after the final version of ICMR-MINDS CDSS was ready for use. This happened before the first training of HCPs in the block.
Step 6: Who participated in the decision?	Deputy District Civil Surgeon cum Nodal Officer for National Mental Health Programme, Faridabad District; HCPs. The decision was taken collaboratively.
Step 7: How widespread is the modification?	The modification pertains to the whole of the public health system in Faridabad district and will be implemented for the remainder of the project.

The AIIMS, New Delhi Project team was also part of the discussions around the modifications for the strategy. AIIMS, All India Institute of Medical Sciences; HCP, Health care professionals; ICMR, Indian Council of Medical Research.

The eighth and ninth adaptations were regarding shadowing other experts and visiting other sites. Initially, it was planned that observation sessions would be arranged at facilities where intervention is being implemented directly to observe the process. The context (format and setting) of the strategies was modified, and the HCPs were requested to share their experience in their monthly meetings. This strategy seeks sought to facilitate informed discussions about the change and its implications amongst early adopters of the interventions and their peers. The modification was made due to time and other logistical constraints and other competing responsibilities that would hinder the execution of observation visits and meetings across facilities (Table 7).

**Table 7:** Modifications to implementation strategies 8 and 9 for integration of screening and management of mental disorders, including substance use disorders (MSUD)care within the existing other non-communicable diseases (NCD) care in Faridabad district of Haryana state based on FRAME-IS.

Step 1: Strategy	Strategies: Shadow other experts and Visit other sites Specific action(s): Arrange observation sessions at facilities where intervention is implemented to obtain direct observation
Step 2: What is modified?	The context (format and setting- how and where learning from others happened) of the strategy has been modified. Instead of arranging visits to other health facilities, HCP shared their experiences during monthly meetings.
Step 3: Nature of modification	Substitution: organising monthly meetings with HCPs instead of organising site visits to shadow experts. The setting and the modality of implementation were modified. Rather than bring the HCPs to the facilities where the interventions had been rolled out, the HCPs from such facilities were requested to share their experience in monthly meetings of the HCPs.
Step 4: Goal and rationale	Goal- to ensure that the early adopters of the interventions had opportunities to share the change and its impact with their peers. Rationale- it was not feasible to arrange observation visits and meetings for HCPs across facilities due to logistical issues such as time constraints and other competing responsibilities. The shift in the strategy offers an opportunity to achieve the desired goal of the implementation strategy by increasing the feasibility by reducing time constraints and logistical demands of individual shadow visits. It also offered a social incentive to the early adopters. Level: organisational level (time constraints, competing responsibilities)
Step 5: When was the modification initiated?	The modification was made during the implementation phase. The modification was initiated from the second month of rolling out the model when the logistical constraints were realised.
Step 6: Who participated in the decision?	Deputy District Civil Surgeon cum Nodal Officer for National Mental Health Programme, Faridabad District; Roster in-charge of HCPs. The decision was taken collaboratively.
Step 7: How widespread is the modification?	The modification pertains to the whole of the public health system in Faridabad district. and will be implemented for the remainder of the project.

The AIIMS, New Delhi Project team was also part of the discussions around the modifications to the strategies. AIIMS, All India Institute of Medical Sciences; HCP, Health care professionals; ICMR, Indian Council of Medical Research.

### New implementation strategies introduced

In addition to modifying the nine original strategies, the “alter incentive/allowance structures” implementation strategy (as per ERIC taxonomy) was introduced after the implementation of the Model M0. This has also been described using the FRAME-IS framework in Table 8.

**Table 8:** Newly introduced implementation strategy after the implementation of the Model M0.

Step 1: Strategy	Alter incentive/allowance structures
Step 2: What is modified?	Context – Addition of a new strategy. The strategy introduced incentive/allowance structures for healthcare professionals.
Step 3: Nature of modification	Strategy – To introduce incentives to the HCPs for adoption of the interventions. Specific action- Social incentives were introduced in the form of appreciation certificates, token gifts, and verbal acknowledgement that were presented to the early adopters in the presence of their peers during the monthly meetings at the CHC/district hospital in Faridabad and during in- person interaction with the respective HCP at their workplace.
Step 4: Goal and rationale	Goal – The goal was to facilitate the adoption of the interventions. Rationale – There were no provisions for any other incentives or rewards for the HCPs for the adoption of the interventions. The adoption of interventions added to the existing responsibilities of the HCPs. The introduction of social rewards incentivised the extra efforts made by the HCPs.
Step 5: When was the modification initiated?	The modification was made during the implementation phase. The strategy was introduced after around 10 weeks from the start of the implementation.
Step 6: Who participated in the decision?	Deputy District Civil Surgeon cum Nodal Officer for National Mental Health Programme, Faridabad District; AIIMS New Delhi Project Team
Step 7: How widespread is the modification?	We intend to use the strategy for the remainder of the project.

The AIIMS Project team was also part of the discussions around the introduction of new strategy.

This new implementation strategy was to alter incentive/allowance structures for healthcare professionals. The modification was categorised under context (other strategies). We presented social incentives in the form of appreciation certificates, token gifts and verbal acknowledgement to early adopters in the presence of their peers during the monthly meetings at the respective CHC/district hospital in Faridabad and during in-person interaction with the respective HCP at their workplace. The aim was to incentivise the HCPs to adopt the intervention. The adoption of interventions increased the existing responsibilities of HCPs. While no other incentives or rewards were provided, the introduction of social rewards aimed to acknowledge and motivate their additional efforts. The decision was made in consultation with the Deputy District Civil Surgeon cum Nodal Officer for National Mental Health Programme, Faridabad District. It was adopted for the whole Tigaon block, and was aimed to implement it for the remainder of the project.

### Categorisation of modifications

Of the total of nine modifications, three were categorised as proactive modifications and six as reactive modifications. In addition, eight modifications were categorised as planned modifications and one as unplanned modification (Table 9).

**Table 9:** Categorisation of modifications made to the implementation strategies.

Modification made to	Planned/unplanned modifications	Proactive/reactive modification
Strategy: Revise professional roles/Create new clinical teams Specific action(s): Develop a proposal for new staffing arrangements (number, roles)	Planned (for CHOs) Planned (for Psychiatrist)	Reactive (for CHOs) Proactive (for Psychiatrist)
Strategy: Facilitate relay of clinical data to providers Specific action(s): Assess existing/non-existing Health Information Technology (HIT) systems	Unplanned	Reactive
Strategy: Promote network weaving Specific action(s): Organise meetings, collaborative learning sessions to discuss issues and updates, share experiences, problem-solving, and support one another	Planned	Reactive
Strategy: Intervene with patients/consumers to enhance uptake and adherence Specific action(s): Identify relevant stakeholders; Map stakeholders by their influence, power, authority, and importance in relation to implementation using the Mendelow’s matrix; Activate stakeholder groups	Planned	Proactive
Strategy: Use mass media Specific action(s): Spread information about the implementation using IEC material	Planned	Proactive
Strategy: Conduct ongoing training Specific action(s): Conduct induction, follow up and refresher trainings on use of interventions	Planned	Reactive
Strategy: Shadow other experts/Visit other sites Specific action(s): Arrange observation sessions at facilities where intervention is implemented to obtain direct observation	Planned	Reactive
Introduction of “Alter incentive/allowance structures” strategy	Planned	Reactive

## Discussion

This article describes the process and outcomes of the modifications to the implementation strategies that were created as part of the Model 0 (M0) (subsequent interim models M1, M2, M3…) for integration of MSUD care into the existing NCD care in the public health system in the Faridabad district of Haryana state in India. The adaptations made to the original strategies in response to on-the-ground challenges and contextual realities were systematically tracked and reported using FRAME-IS.

One of the key learning from this study is the inherently dynamic nature of implementation. The original set of implementation strategies was co-created with the stakeholders using the ERIC taxonomy (24). However, implementing in real-world settings required flexibility. Several unanticipated challenges were encountered during implementation. These included delays in HCP appointments, logistical barriers to travel, scheduling conflicts, and low feasibility of formal engagement mechanisms.

The FRAME-IS framework was used for the documentation and reporting of modifications in this study (18). A structured and transparent method for recording was provided by FRAME-IS. It helped document what was modified, why it was modified, who was involved, and when and how the changes took place. FRAME-IS has increasingly been used to track and analyse strategy modifications in clinical trials and system-level improvement efforts (20–22, 26, 27). Use of FRAME-IS helped go beyond the traditional approach of outcomes-focused evaluations. We were able to document the process in itself.

The participatory action research (PAR) approach enabled continuous engagement with project field staff and other stakeholders. Daily log documentation, training reports, data form digital portal, and periodic review meetings created feedback loops between implementers and researchers. This resulted in alignment of the implementation strategies with local needs. In addition, it enhanced the relevance of the strategies used. Such an iterative approach has been shown to contribute to more sustainable change in previous research (12, 17).

A total of nine substantive (core-function) modifications were made. Additionally, one new strategy was introduced during the course of implementation of model M0 and its subsequent iterations (Model M1, M2, M3…). Also, each modification was categorised as either proactive or reactive, and as either planned or unplanned. This categorisation helped highlight the nature of the adaptations and the underlying drivers. It was found that reactive and unplanned modifications were generally associated with immediate administrative issues or operational barriers. On the other hand, proactive and planned modifications were based on stakeholder feedback and early implementation learnings.

The study aimed to maintain the fidelity of the proposed implementation strategies. Although changes were made to the form of the strategies, the focus was to ensure that the core functions remained unchanged. For example, in the case of the strategy to promote network weaving, the original plan to conduct formal collaborative sessions was replaced with informal peer interactions due to scheduling conflicts. While the delivery modality changed, the underlying purpose remained intact. Similarly, changing the training venue from AIIMS New Delhi to local health facilities ensured the intent of capacity-building. At the same time, it enhanced feasibility and reduced the demands on the HCPs. It has been reported previously that adaptations that alter the delivery form but retain the core function and theoretical underpinnings of a strategy can enhance contextual fit without compromising effectiveness (28).

Innovations were introduced in the form of reallocation of tasks to ANMs and nurses in the absence of CHOs, the shift from mass media to localised awareness sessions, identification of psychiatrists from CRHSP Ballabgarh to provide referral services, the use of ASHAs to spread awareness on MSUD during home visits, and the leveraging of existing monthly meetings for peer sharing. These are examples of locally relevant solutions that were feasible and effective. Moreover, these modifications were the result of a collaborative process, thereby highlighting the importance of a participatory approach. Engaging with health system actors and building in mechanisms for continuous feedback and adaptation created a locally relevant and context-sensitive implementation framework. The original plan to include patients/service users and caregivers in implementation workgroups was found to be inconvenient for many due to logistical barriers. Instead, the modified specific action of using IDIs to capture their perspectives was developed in consultation with them.

Model Mx will be the final implementation model developed through systematic refinement of the prototype Model M0 to support integration of screening and management of MSUD into routine NCD care in public health facilities in Faridabad district, Haryana. The model will be shaped through repeated field testing and structured adaptation guided by the CFIR, Implementation Mapping, Intervention Mapping, the Implementation Research Logic Model, and RE-AIM. The aim will be to retain the core functions of integrated MSUD–NCD care while modifying delivery to match the ground reality.

### Implications for policy and practise

The findings of this study have certain implications for policy and practise. These are particularly relevant in the context of LMICs and MSUD care. First, frameworks such as FRAME-IS should be integrated into large-scale health implementation research. This enables proactive anticipation of potential modifications and creates mechanisms for documentation. Second, participatory frameworks that allow continuous interaction between different actors (stakeholders) and researchers should be developed. This is in keeping with a growing mental health and addiction literature that emphasises co-design and lived experience as central to improving service relevance and acceptability (29, 30). Structured inclusion of lived experience has shown to strengthen service responsiveness and reduce stigma in high income countries. This is applicable even when the final decision-making authority remains within health systems (31, 32). Third, capacity-building strategies should take into account the logistical constraints. This means that training design should be flexible and aligned with the operational realities of HCPs. Policy and large-scale programmes could benefit from incorporating guidance on such decentralised models to increase feasibility. The digital platform and dashboard created as part of the initiative are aimed to increase the use of digital interventions in MSUD care delivery. This is not only for the purpose of service delivery, but also for supervision and feedback purposes. This remains limited in LMICs (33). Strategies that relied on specific cadres of HCPs (such as CHOs or psychiatrists) were found to be vulnerable to staffing shortages. Substitutions (such as entrusting ANMs and nurses to take on additional roles) helped with continuing service delivery. Similarly, replacing formal mass media campaigns with localised awareness sessions ensured timely dissemination of information. Fourth, the use of social incentives such as public recognition of early adopters was used as a low-cost strategy for motivating HCPs. Finally, this study also highlights the importance and feasibility of structured documentation of implementation adaptations to inform future scale-up.

### Limitations

The FRAME-IS framework allowed for comprehensive documentation of modifications to the implementation strategies. However, there are certain limitations of this work. First, the study was carried out in a single district during the early phase of implementation. As such, the generalizability of some findings may be limited, especially in settings with different health system structures. However, Faridabad’s mix of urban and rural areas and its classification as a weaker-performing district do provide valuable insights into implementation under challenging conditions. In addition, all the public health facilities across all levels of care, including AAM-SC, AAM-PHC, CHC, Sub Divisional (District) Hospital, and the District Hospital (more than 116 in total) are part of the study. Second, great care was taken to document changes in real time. However, the boundaries between planned and unplanned modifications are not well defined and these may overlap. Future work may benefit from cross-site comparisons to refine these categorisations further. Finally, we introduced a new strategy in the form of social incentives. Such additions are expected to enhance responsiveness. Although, such additions may complicate fidelity assessment. Careful documentation and evaluation are required to assess the cumulative impact of such strategies on implementation outcomes. Also, the less extensive refinement (form-preserving) modifications made to the other strategies were not reported.

Despite these limitations, there are multiple strengths of this work. By providing a detailed account of the adaptations made during the initial phase of implementation, this study contributed to the evidence base on how implementation strategies can be refined in real-world settings in LMICs in South East Asia and other regions. This study also highlighted the importance of structured frameworks in capturing the complexities of strategy modification and in guiding future replication and scale-up efforts. To the best of our knowledge, this is the first published attempt at using FRAME-IS for integration of MSUD care into the existing NCD care framework as part of a national programme.

## Conclusion

This study provides a detailed account of how implementation strategies were pragmatically modified to support the integration of MSUD care into existing NCD services within a resource-constrained public health system in Faridabad district in Haryana. The nature, rationale, scope, and timing of these modifications across nine implementation strategies were documented.

The findings suggest that real-world implementation requires adaptations to address contextual factors. These include human resource limitations, administrative barriers, and logistical challenges. Also, it offers an opportunity to leverage on the local facilitators and resources. The adaptations that were made ranged from revisions in professional roles to the delivery of training and use of informal peer networks. These were made collaboratively with the stakeholders. This participatory approach enhanced feasibility and acceptability. It also preserved the core functions of the original strategies.

These modified implementation strategies contributed to the iterations of Model M0 (and subsequent iterations M1, M2, M3…) and development of Model Mx. These strategies reflect a more contextually grounded and system-responsive set of implementation strategies that are now positioned for scale-up and sustainability. The use of FRAME-IS enabled transparent reporting. It can also serve as a practical guide for other implementation efforts on MSUD.

Systematic documentation of strategy modifications should be emphasised as part of the future implementation research. This is relevant in the context of all regions of the world. This shall help build a cumulative evidence of implementation adaptation in the context of MSUD.

## Data Availability

The raw data supporting the conclusions of this article will be made available by the authors on a reasonable request to the corresponding author.
